# Full-term abdominal extrauterine pregnancy complicated by post-operative ascites with successful outcome: a case report

**DOI:** 10.1186/1752-1947-7-10

**Published:** 2013-01-09

**Authors:** Gwinyai Masukume, Elton Sengurayi, Alfred Muchara, Emmanuel Mucheni, Wedu Ndebele, Solwayo Ngwenya

**Affiliations:** 1 Department of Obstetrics and Gynaecology, Mpilo Central Hospital, Bulawayo, Zimbabwe; 2 Department of Anaesthesia, Mpilo Central Hospital, Bulawayo, Zimbabwe; 3 Department of Paediatrics, Mpilo Central Hospital, Bulawayo, Zimbabwe

## Abstract

**Introduction:**

Advanced abdominal (extrauterine) pregnancy is a rare condition with high maternal and fetal morbidity and mortality. Because the placentation in advanced abdominal pregnancy is presumed to be inadequate, advanced abdominal pregnancy can be complicated by pre-eclampsia, which is another condition with high maternal and perinatal morbidity and mortality. Diagnosis and management of advanced abdominal pregnancy is difficult.

**Case presentation:**

We present the case of a 33-year-old African woman in her first pregnancy who had a full-term advanced abdominal pregnancy and developed gross ascites post-operatively. The patient was successfully managed; both the patient and her baby are apparently doing well.

**Conclusion:**

Because most diagnoses of advanced abdominal pregnancy are missed pre-operatively, even with the use of sonography, the cornerstones of successful management seem to be quick intra-operative recognition, surgical skill, ready access to blood products, meticulous post-operative care and thorough assessment of the newborn.

## Introduction

Advanced abdominal (extrauterine) pregnancy (AAP) is defined as a fetus living or showing signs of having once lived and developed in the mother’s abdominal cavity after 20 weeks of gestation [1].

A hospital-based study from Zimbabwe showed an AAP incidence of one in 9500 total deliveries and one in 60 ectopic pregnancies [2].

AAP can be classified as being primary or secondary [3]. Primary AAP occurs when the fertilized ovum implants directly into the peritoneal cavity; primary AAP is the less common type. Secondary AAP occurs when the fertilized ovum first implants in the fallopian tube or uterus and then due to fimbrial abortion or rupture [4,5] of the fallopian tube or uterus the fetus comes to live and develop in the mother’s abdominal cavity. Ruptured tubal ectopic pregnancies account for the majority of AAP.

Ovarian, tubal and intraligamentary pregnancies are excluded from the definition of AAP.

Maternal and fetal morbidity and mortality are high with AAP [1-5].

Making the diagnosis of AAP is difficult even with the ubiquitous use of sonography during pregnancy [6]. Most cases of AAP are missed and are only diagnosed during surgery [1]. Operative and post-operative management of AAP is also difficult [7].

In addition, all degrees of pre-eclampsia may occur more frequently with AAP [7-13] presumably due to inadequate placentation during early pregnancy [14] as a result of the commonly abnormally sited placenta found in AAP. Pre-eclampsia and its serious complications are leading causes of maternal and perinatal morbidity and mortality.

We report a case of full-term AAP without maternal or fetal death.

## Case presentation

A 33-year-old primigravid African woman who had been pregnant for 40 weeks and three days dated from the first day of her last normal menstrual period presented to our unit with a three-day history of continuous pain around the umbilical area. She did not have any other complaints.

A review of her antenatal records revealed that she had sought medical attention at her local clinic for lower abdominal pain at eight weeks and four days and once again at 14 weeks and two days of gestation. Both episodes of lower abdominal pain were managed conservatively.

Throughout the pregnancy the patient continued to experience on and off abdominal pain which became better as the pregnancy progressed.

She denied any per vaginal bleeding during her pregnancy.

Of note, the patient said that her mother had developed hypertension when she was pregnant.

The results of her serologic tests for human immunodeficiency virus as well as rapid plasma reagin were negative at the antenatal clinic. Her hemoglobin concentration at 22 weeks of pregnancy was 10.7g/dL. Methyldopa and nifedipine were commenced for pregnancy-induced hypertension from 22 weeks of gestation. During antenatal care visits at 26 weeks and three days, 30 weeks and three days, 34 weeks and four days, and 37 weeks and three days she was normotensive. At 38 weeks and four days her blood pressure was 150/100mmHg.

The patient had an ultrasound scan done at 33 weeks and three days at a local clinic which showed a single viable breech intrauterine pregnancy with a left lateral upper segment placenta, slightly reduced liquor volume and some small free fluid in the hepatorenal area. The estimated gestational age by scan was 34 weeks and two days. From the time of this ultrasound scan, the presentation by clinical examination which had previously been documented as cephalic was then documented as breech.

Besides the on and off abdominal pain, her pregnancy had been uneventful until she developed periumbilical pain prompting her to present to our unit.

On examination her blood pressure was 140/90mmHg and other vital signs were within normal limits. Her body mass index (her weight in kilograms divided by the square of her height in meters) was 27. A breech presentation was the remarkable finding on obstetric examination. Admission and urgent delivery by cesarean section was advised because of the breech presentation and suspected pre-eclampsia; the patient agreed and she gave her informed consent.

She continued taking antihypertensives. During her admission, several of her blood pressure readings were in the severe range (≥160/110mmHg). At 40 weeks and five days of pregnancy our patient had a cesarean section performed via a Pfannenstiel incision by a junior member of the obstetric team. On entering the ‘parietal peritoneum’ a sudden gush of meconium-stained liquor issued forth. The fetal back was visualized. Delivery of the baby was done, the umbilical cord was clamped and cut and part of the placenta was removed. On recognition of the unusual circumstances, artery forceps were used to arrest bleeding from the remaining placenta and then senior help was sought. The cesarean section which was being done under spinal anesthesia was continued under general anesthesia (the patient was intubated) because severe hemorrhage was anticipated and also to minimize patient anxiety.

AAP was diagnosed by the next most senior doctor who in turn sought assistance from the consultant.

**Figure 1:**
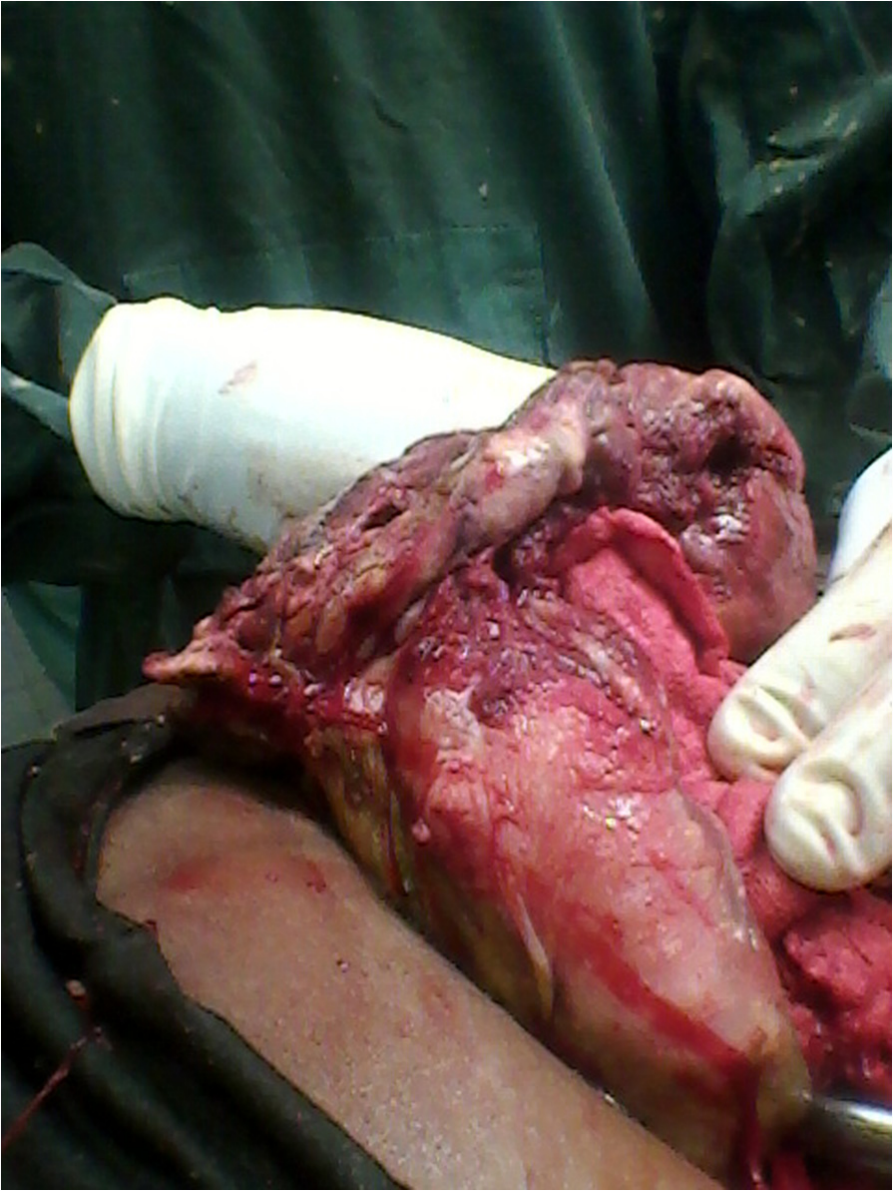
**Placenta and membranes adherent to loop of bowel (anterior view).** Note linea nigra.

**Figure 2:**
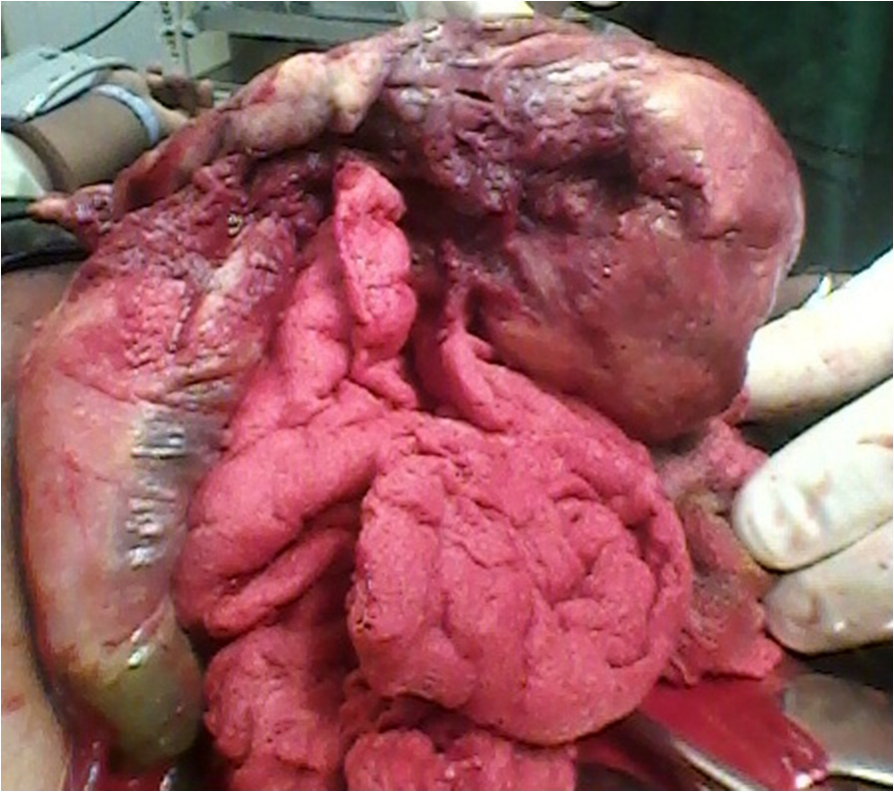
Placenta and membranes adherent to loop of bowel (posterior view).

When the consultant arrived, the patient’s placenta was noted to be predominantly attached to her small bowel (Figures 1 and 2), large bowel and the superior surface of her urinary bladder. After assessment of the placental attachment site, a decision was made to remove the remaining placenta. There were large bleeders on the placental bed which was thick enough to allow figure of eight sutures to be used without compromising nearby organs.

The meconium-stained bowel was cleaned with normal saline; no obvious bowel defects were noted. Part of the membranes attached to the small and large bowel and bladder were left *in situ*. The uterus was palpable and about the size of a uterus at 10 weeks of pregnancy beneath the sac. Estimated blood loss was 1.5liters. Our patient received three units of packed red blood cells.

The baby girl delivered weighed 2850g and had Apgar scores of eight and 10 at one minute and five minutes respectively. Besides slight cranial asymmetry the examination of the newborn baby was unremarkable. The baby’s head circumference was 32cm (5th centile). By day 5 post-laparotomy the mother could mobilize fully, eat, drink and had normal bowel motions and micturition. The baby was breast feeding and eliminating well. Of note, the mother’s blood pressure normalized post-delivery; however, the results of a urine dipstick test showed that she had proteinuria (3+ = 3g/L).

On day 5 post-delivery, an ultrasound scan showed a slightly bulky uterus measuring nine cm in length not consistent with a post-delivery uterus. Notably, the scan showed ascites with fibrinous strands and internal echoes.

The patient requested discharge and she was discharged on day 5 after delivery on iron and folate tablets. During her stay, she was counseled on AAP and future care.

On day 9 after her laparotomy our patient was re-admitted with a three-day history of vomiting after meals, abdominal pain and distension; she was passing stool and flatus normally.

A physical examination revealed a grossly distended abdomen. A repeat ultrasound scan showed gross ascites. Functional intestinal obstruction secondary to increased intra-abdominal pressure from the gross ascites was diagnosed. Serial abdominal ascitic tapping for symptomatic relief, intravenous crystalloid and antibiotics as well as low molecular weight heparin were commenced.

On day 15 post-surgery, 5700mL of a dark brown fluid was drained at one go from the urinary catheter and her abdominal distension subsided. Over the following days, her condition was monitored.

The patient and her baby were discharged from hospital on hematinics with no complaints 26 days after the birth of her baby.

Five weeks following surgery, the patient and her baby were apparently doing well.

At about six months of age, the infant’s growth and development were seemingly normal. The infant’s head circumference was 40cm (5th centile); the cranial asymmetry was barely noticeable.

At about six months after delivery, the mother had no complaints and her physical examination was unremarkable. Her blood pressure was 160/93mmHg; she had hypokalemia (3.3mmol/L, reference range (RR) 3.6 to 5.0mmol/L) and hyponatremia (124mmol/L, RR 135 to 145mmol/L). Her chloride was 101mmol/L, RR 101 to 111mmol/L and bicarbonate, 15mmol/L, RR 21 to 31mmol/L.

The mother’s laboratory blood investigations are shown in Table 1.

**Table 1:** Laboratory data

**Variable**	**Reference range**	Day 1*	**Day 9**	**Day 12**	**Day 16**	**Day 19**	Approximately 6 months**
White cell count (× 10^9^/L)	6–14	10.89	16.72	13.2	11.59		2.82
Hemoglobin (g/dL)	11.5–15.5	8.7	9.4	8.6	10.4		12.1
Platelets (× 10^9^/L)	150–400	118	642	725	576		214
Mean cell volume (fl)	80–95	87.5	89.6	93.8	90.3		85.0
Mean cell hemoglobin (pg)	27–34	29.5	28.8	26.6	29		28.9
Mean cell hemoglobin concentration (g/dL)	30–35	33.7	32.2	28.4	32.1		34.0
Urea (mmol/L)	3.2–6.7	7	16.8	21		14.9	3.8
Creatinine (micromol/L)	53–115	75	347	377		316	40
Aspartate aminotransferase (IU/L)	10–42	50		26			
Alanine aminotransferase (IU/L)	6–28	20		12			
Direct bilirubin (micromol/L)	0–3	18		3			
Albumin (g/L)	23–38	18		22			
Total protein (g/L)	67–82	49		77			

There is a normochromic normocytic anemia consistent with acute blood loss with a possibly reactive thrombocytosis. The initial thrombocytopenia could have been caused by pre-eclampsia. Raised urea and creatinine are probably secondary to hypovolemia due to loss of fluid into the peritoneal cavity leading to pre-renal acute kidney injury. Aspartate aminotransferase is usually raised after cesarean section. Conjugated hyperbilirubinemia is attributed to intra-operative hemorrhagic shock. Because albumin is a negative acute phase reactant, the trauma of surgery may have contributed to the hypoalbuminemia which improved over time post-operatively. Hypoproteinemia was probably due to proteinuria and also possibly resulted from the period of starvation before and after surgery. Leucopenia at six months did not escape our attention. The patient was rhesus D positive.

* Days post-surgery.

** Different reference ranges may apply; there was no proteinuria.

## Discussion

In retrospect, this patient’s history, physical examination and radiologic findings were in line with a diagnosis of AAP. Lower abdominal pain at eight weeks and four days of gestation coincides with the peak period of ruptured ectopic pregnancies [15].

It is probable that the suspected tubal ectopic pregnancy ruptured and then subsequently re-implanted in the peritoneal cavity at this time.

Most cases of AAP have an abnormal lie [1]; our patient had a breech presentation.

Free maternal intraperitoneal fluid as was noted on our patient’s ultrasound scan at 33 weeks and three days has been noted to be a feature of AAP [3,4,7]. In addition, reduced amniotic fluid [16] is another feature of AAP which was also noted on our patient’s ultrasound scan.

As mentioned earlier, pre-eclampsia is not uncommon with AAP and this could have further led one to suspect AAP. However, a lack of resources to confirm the diagnosis, a family history suggestive of the disease and nulliparity which are both risk factors for developing pre-eclampsia, as well as an ultrasound scan which apparently showed an intrauterine pregnancy all confounded issues.

Taken together, the history of lower abdominal pain in early pregnancy, continued abdominal pain during pregnancy, an abnormal lie, free maternal intraperitoneal fluid and reduced amniotic fluid on sonography could have all helped clinch the diagnosis of AAP. However, the diagnosis of AAP remains difficult.

We attribute the patient’s continuous periumbilical pain at presentation to irritation of the visceral peritoneum somewhere between the jejunum and transverse colon, originating from the placental attachment site. Pain from the jejunum to transverse colon is felt around the umbilicus (referred pain) [17].

The central controversy in managing AAP regards management of the placenta [1].

Removing the placenta can result in catastrophic hemorrhage [3] and damage to adjacent structures, whereas leaving it *in situ* may lead to secondary hemorrhage, abscess formation, adhesions, coagulopathy, continued pre-eclampsia, failure of lactogenesis [18], a need for second surgery and a need for longer follow-up among other complications.

In our case, we had ready access to immediate blood transfusion which most patients with AAP receive [1]. Where blood transfusion and intensive care are not readily available, leaving the placenta *in situ* has been recommended [19]. Placental removal is done after assessing for safety and feasibility of removal.

The finding of slight cranial asymmetry in the newborn was not unusual because about 20% of AAP infants have malformations or deformations [20]. We ascribe this cranial asymmetry to skull compression due to the lack of the protective myometrial wall and perhaps the reduced amniotic fluid noted on sonography. Although perinatal morbidity and mortality are high with AAP, normal development of the infant at follow-up as in our case is possible although very rare [19].

We speculate that the ascites was due to probable transudation or exudation of fluid from the site at which the placenta was attached. We are of the opinion that the fluid drained from the urinary catheter on day 15 post-surgery came from the peritoneal cavity through an abnormal communication which developed due to increased intra-abdominal pressure where the placenta was adherent to the urinary bladder (a point of weakness). We lacked the financial resources to do the necessary tests to ascertain the origin and nature of this fluid.

We are aware of what may seem like deficiencies in management; we work in a low-income country. Pathologic examination of the placenta would have been useful [21].

This patient is probably at higher risk of having a future ectopic pregnancy [22], cardiovascular and renal disease [14]. In order to optimize the patient’s next pregnancy, she was advised to attend pre-conception counseling. She was also advised of the need to have close follow-up of her current baby.

The mother’s constellation of hypertension, hypokalemia and hyponatremia at about six months after delivery raises the possibility of a hyperreninemic state which requires further investigation to clarify and manage [23]. The significance of a hyperreninemic state in relation to AAP is uncertain.

It is very rare to have a live AAP proceeding to term and being successfully managed [4]. It is because of this we report this case.

## Conclusion

Because most diagnoses of AAP are missed pre-operatively even with the use of sonography, the cornerstones of successful management seem to be quick intra-operative recognition, surgical skill, ready access to blood products, meticulous post-operative care and thorough assessment of the newborn.

## Consent

Written informed consent was obtained from the patient for publication of this case report and any accompanying images. A copy of the written consent is available for review by the Editor-in-Chief of this journal.

## Competing interests

The authors declare that they have no competing interests.

## Authors’ contributions

SN was the patient’s primary physician. GM wrote the first draft of the article. ES, AM, EM, WN and SN revised the manuscript making important intellectual contributions. All authors read and approved the final version of the manuscript.
